# Systematic Review of Herbal Tea (a Traditional Chinese Treatment Method) in the Therapy of Chronic Simple Pharyngitis and Preliminary Exploration about Its Medication Rules

**DOI:** 10.1155/2019/9458676

**Published:** 2019-09-19

**Authors:** Chengxian Li, Fucang Wu, Weiling Yuan, Qi Ding, Min Wang, QingQing Zhang, Ju Zhang, Jingyu Xing, Shang Wang

**Affiliations:** ^1^Tianjin University of Traditional Chinese Medicine, Tianjin 301617, China; ^2^Beijing University of Chinese Medicine Dongfang College, Beijing 065001, China

## Abstract

**Background:**

Chronic simple pharyngitis (CSP) is a common clinical chronic respiratory inflammation with persistent and intransigent symptoms. We analyzed the clinical data to find the evidence that herbal tea, a traditional Chinese medicine treatment in China, could improve the symptoms of CSP patients in a simple way.

**Methods:**

We systematically reviewed the clinical data of randomized controlled treatments from April 2019 and evaluated the results using the improved Jadad scale and the Cochrane bias risk assessment tool. RevMan 5.3 software was used for chart analysis. In addition, we used Excel to conduct frequency statistics on Chinese herbs from included articles and analyze its medication rules.

**Results:**

Among the collection of 161 articles, 6 RCTs published in Chinese journals were included in this review. The methodological quality of the treatments was low, and most of them only provide diagnostic criteria. Inclusion and exclusion criteria were not specified, and none of the 6 RCTs used the blind method on the result evaluator. Furthermore, only one RCT evaluated the baseline level variance. For these reasons, we did not make a network meta-analysis.

**Conclusions:**

The traditional Chinese herbs involved in herbal tea did have ingredients to alleviate CSP symptoms. However, our research showed that the current research could not draw any credible conclusions on the curative effect of herbal tea, which indicated that the overall level of TCM clinical research needs to be improved to evaluate the efficacy of herbal tea.

## 1. Introduction

Chronic simple pharyngitis, a kind of chronic pharyngitis, is a common disease in the otolaryngology field. It is characterized by diffuse chronic inflammation of the pharyngeal mucosa, submucosa, and lymphoid tissue and is also a part of chronic inflammation of the upper respiratory tract. Its incidence rate is from 10% to 20% in pharyngeal diseases. Among the clinical manifestations, which primarily include pharyngeal pain, foreign body sensation, itching, coughing, etc., the reflex nausea and vomiting while brushing teeth in the morning has seriously lowered the quality of life of patients [1]. Modern medicine believes that the occurrence of this disease is related to a variety of factors, of which the repeated attack of acute pharyngitis is the main cause. Upper respiratory tract lesions adjacent to the pharynx, climate and regional environmental changes, occupational factors, systemic factors, and allergic factors can also cause the disease [2, 3]. In addition, an Indian scholar, Kumari, reported that group A streptococcal pharyngitis (GASP) was one of the culprits of chronic pharyngitis [4]. There are also reports indicated that the virus was detected in the pharynx of patients with CSP, and the pathogenesis was related to viral infection which mainly referred to EB virus and adenovirus. In the United States, over 11 million people are suffering from pharyngitis every year. Unsurprisingly, due to various factors such as Chinese diet and environmental pollution, the incidence of chronic pharyngitis in China is also high [5–7]. According to the report of 2012, one-third of the patients in the otolaryngology clinic suffered from chronic pharyngitis [8].

Conventional treatment of CSP chiefly includes antibiotics and aerosol inhalation. Antibiotics may lead to drug resistance, and the abuse of it can result in dysbacteriosis of the throat and consequently give rise to double infection, in which the patient's condition may even deteriorate further. The aerosol inhalation treatment tends to be repeated more than once and still cannot treat CSP effectively [9]. Considering the current curative effect, it is imperative to find another solution.

Herbal tea, a traditional therapy in China, refers to a kind of brewed tea along with single or multiple flavors of Chinese herbs [10]. It has a long history in the development of traditional Chinese medicine and is generally believed to be beginning in the Tang dynasty, flourishing in the Song dynasty, and matured in the Qing dynasty. Chen Cangqi (687–757 A.D.), a medical scientist in the Tang dynasty, first put forward the concept of “herbal tea therapy” in his famous medical book *A Supplement to Materia Medica* and said that tea was the remedy for all kinds of diseases. By the time of Qing dynasty, herbal tea had been fully developed and highly praised; Chen Keji, an academician of the Chinese Academy of Sciences, had found at least 8 kinds of herbal tea in the original medical files of the emperor in the Qing dynasty, including Nectar tea, *Ganoderma lucidum* tea, Shenqu tea, *Sophora japonica* tea, *Chrysanthemum indicum* tea, *Stereulia lychnophora* tea, *Nelumbo nucifera* tea, and *Isatis indigotica* tea, which was recorded in *Imperial Medicaments*—medical prescriptions written for Empress Dowager Cixi and Emperor Guangxu with commentary [11, 12]. Herbal tea is effective in the treatment of chronic diseases and is convenient and economical enough for long-term use [13].

However, herbal tea has drawn little worldwide attention and been lack of systematic reviews on its curative effects on CSP until now. In addition, there was no specific and unanimous composition of herbal tea for CSP treatment in current studies. Based on these questions, the article was written to provide a preliminary image on herbal tea in the treatment of CSP.

## 2. Materials and Methods

### 2.1. Types of Studies

We included randomized controlled trials (RCTs) only.

### 2.2. Search Strategy

In order to find out all the literature, an extensive search was performed. We searched the Cochrane Library (*The Cochrane Library* 2019, Issue 4; https://www.cochranelibrary.com), PubMed (https://www.ncbi.nlm.nih.gov/pubmed/), Web of science (http://apps.webofknowledge.com), EBSCO (http://search.ebscohost.com), Elsevier (https://www.sciencedirect.c-om/), Springer (http://link.springer.com/), CNKI (*China National Knowledge Infrastructure*, http://www.cnki.net/), Wanfang (http://www.wanfangdata.com.cn/), and VIP (*Chongqing VIP Information Co., Ltd*, http://www.cqvip.com/) from the year that each database was created to April 2019, using the search strategy “TI = (Chinese medicinal herbal tea or medicinal tea) AND TI = (chronic simple pharyngitis or chronic throat inflammation)” and imposing no language or publication restrictions.

### 2.3. Inclusion and Exclusion Criteria

**Figure 1:**
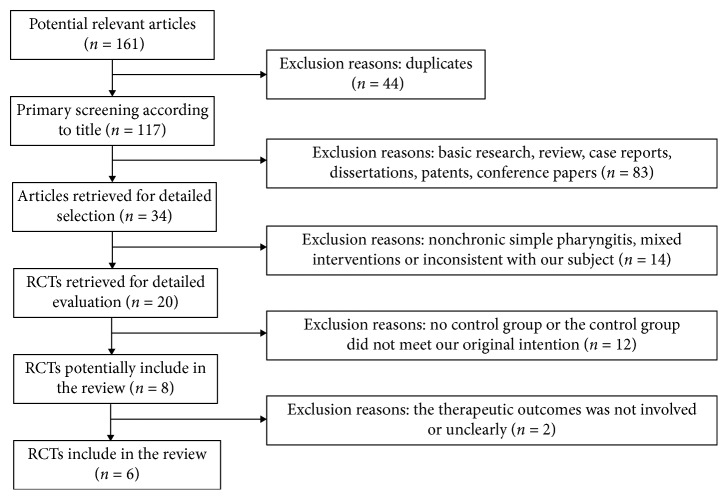
Inclusion process and results of the relevant articles.

The studies were included if they met the following criteria as follows: (1) The study subjects were limited to patients with chronic simple pharyngitis (primarily caused by chronic hyperemia of the pharyngeal mucosa, submucosal connective tissue, and lymphoidal tissue hyperplasia); there was no requirement in terms of age and sex. (2) The study was a randomized controlled trial comparing the treatment group with the control group. (3) The treatment group was treated with only herbal tea, and the control group was treated with conventional medicine. (4) The article had the description of symptoms after treatment. Studies were excluded if they met any of the following criteria: (1) The study subjects were patients mixed with different types of chronic pharyngitis. (2) The study was a review, case report, fundamental research, or patent. (3) Herbal tea was used in combination with other treatments in the treatment group. (4) The article had no record on variations in symptoms. The inclusion and exclusion process is detailed in Figure 1.

### 2.4. Data Extraction

Two review authors (Chengxian Li and Fucang Wu) filtered articles according to the inclusion and exclusion criteria. Then, we extracted the following study characteristics from the included articles (Table 1):

- ^*∗*^Author, Year
- ^*∗*^Number of treatment group and control group
- ^*∗*^Gender composition
- ^*∗*^Age distribution
- ^*∗*^Interventions

### 2.5. Data Analysis (Quality Assessment of Trials Included)

**Figure 2:**
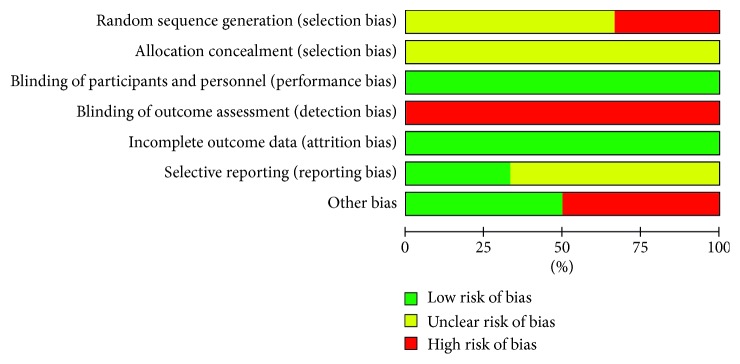
Risk of bias graph: review authors' judgements about each risk of the bias item presented as percentages across all included studies.

**Figure 3:**
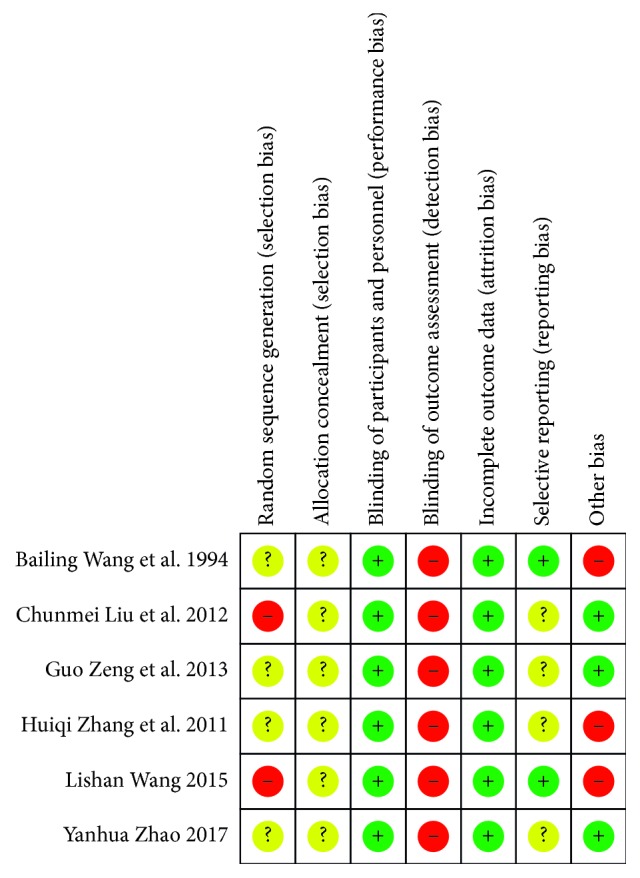
Using the Cochran collaboration's tool for assessing risk of bias about the six studies.

Quality in a systematic review essentially refers to the absence of biases, so we used the modified Jadad scale for evaluating the quality of trials and the Cochrane collaboration's tool (*Cochrane Handbook for Systematic Reviews of Interventions*) for assessing risk of bias (Table 1). Articles were assessed by two reviewers (CXL and FCW) independently, and disagreements were resolved by consultation with the third reviewer (WLY). RevMan 5.3 was used to output chart, detailed in Figures 2 and 3.

## 3. Results

### 3.1. Search Flow

According to the search strategy, we identified 161 potentially relevant studies. By reading titles and abstracts, we excluded 127 studies that were clearly duplicates, basic researches, reviews, case reports, dissertations, patents, and conference papers. After the full-text reading, 14 articles were excluded for including nonchronic simple pharyngitis, containing mixed interventions or being inconsistent with our subject; 12 were excluded for having no RCTs and 2, for unknown interventions effects. The inclusion and exclusion process is detailed in Figure 1.

### 3.2. Participants

Finally, we included six trials involving 650 participants, and all of the articles (the number of participants varying from 68 to 200) were published in China. The age of participants in these trials ranged from 18 to 72 [14–19].

### 3.3. Interventions

The treatment group was treated with herbal tea, while the control group was treated with conventional therapy. We can see details in Table 1.

### 3.4. Methodological Quality and Level of the Efficacy Evaluation

The main biases in a clinical trial come from systematic differences between comparison groups in the selection bias, performance bias, detection bias, attrition bias, reporting bias, and other bias.

#### 3.4.1. Selection Bias

All the studies claimed to be randomized, but Wang [15] and Liu et al. [17] used a wrong random method or pseudorandom method, while other studies did not mention how they produced random sequences. No trial described allocation concealment, so we could not make out whether they used hidden allocation or not.

#### 3.4.2. Performance Bias

The blind method was used both in the treatment group and the control group.

#### 3.4.3. Detection Bias

The blind method was not applied to staff for the evaluation of efficacy.

#### 3.4.4. Attrition Bias

All patients did not fall off, and the data were complete.

#### 3.4.5. Reporting Bias

Wang [15] and Wang and Li [19] had clear criteria for evaluating, while others did not.

#### 3.4.6. Other Bias

Wang and Li [19], in which the effective rate was too high to be convincing, in fact did not compare the baseline information and not make the number of patients in the control group large enough. Zhang and Liu [18] selected a single syndrome (dry lung syndrome due to yin deficiency) from CSP along with asthma; Wang [15] included patients with chronic pharyngitis and chronic hypertrophic pharyngitis; both of them evaluated the efficacy on CSP separately. The rest of the studies seemed to have no other bias.

All the six studies divided the efficacy evaluation into three levels.

1. Recovery: all symptoms disappeared during the treatment.
2. Effective: some changes happened in traditional Chinese medicine signs.
3. Inefficacy: no benefit in any symptoms by the end of the treatment.

### 3.5. Quality of the Evidence Effectiveness

The overall quality of the evidence included is “very low.” Six included papers scored not more than 3 on the improved Jadad scale (1–3 is grouped into low quality, and 4–7 is grouped into high quality), and the results were consistent with the evaluation results of the Cochrane bias risk assessment tool. The studies had glaring omissions and scored low, probably could be blamed for their early publication, in the selection of random methods, allocation concealment, and blinding methods for evaluators. Additionally, it is doubtful whether there was a report bias since none of the six articles had mentioned the occurrence of falling off and no reports of adverse reactions except for Wang [15].

### 3.6. Analysis of Clinical Effect of the Herbal Tea

The effective rate(ER) was the most commonly used measure to evaluate efficacy. In this article, it consisted of two parts: recovery rate and effective rate. Effective rate analysis was based on the improvement of at least one sign of traditional Chinese medicine. In these 6 studies, this measure was reported as effective rate ratio (RR) and calculated as the ratio between the proportion of responders in the treatment group and the proportion of responders in the control group.

### 3.7. Forest Plot

**Figure 4:**
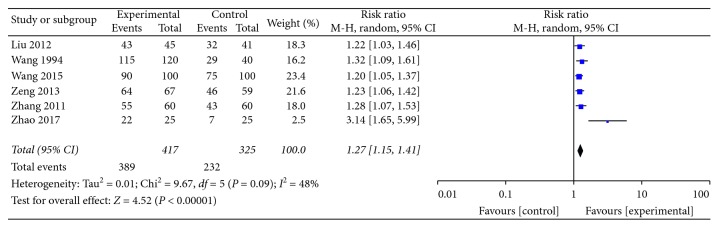
The forest plot of the studies included.

As illustrated in Figure 4, the heterogeneity test *P*=0.09 < 0.1, *I*^2^ = 48%, the overall moderate heterogeneity, the heterogeneity of Zhao [14], and the other 5 experiments are greater, but it was not found in the standard and evaluation rules. There are significant differences with the other 5 groups, which may be related to the scales of the efficacy evaluation of the control group by the experimenter. So, we used the statistical method of risk ratio (M-H, Random, 95% CI). We did not perform a combined analysis for the heterogeneity of the article Zhao [14] and different formulas of herbal tea in other articles. We did not do subgroup analysis either since each formulation of herbal tea was assessed in one study only.

Among the 6 articles included, the efficacy of each test group was stronger than the control group as follows:

  Liu et al. [17], the effect of herbal tea is significantly better than the Yinhuang buccal tablet (RR: 1.22 and 95% CI: 1.03 to 1.46)
  Wang and Li [19], the effect of herbal tea is significantly better than Cao Shan Hu lozenge (RR: 1.32 and 95% CI: 1.09 to 1.61)
  Wang [15], the effect of herbal tea is significantly better than gentamicin injection; dexamethasone injection; chymotrypsin injection; and atomization inhalation (RR: 1.20 and 95% CI: 1.05 to 1.37)
  Zeng et al. [16], the effect of herbal tea is significantly better than cefprozi capsule; atomization inhalation, and Yinhuang buccal tablet (RR: 1.23 and 95% CI: 1.06 to 1.42)
  Zhang and Liu [18], the effect of herbal tea is significantly better than Qingyan pill (RR: 1.28 and 95% CI: 1.07 to 1.53)
  Zhao [14], the effect of herbal tea is significantly better than cefuroxime axetil tablets (C20H22N4O10S) and Yan Li Shuang mouth containing dropping pills (RR: 3.14 and 95% CI: 1.65 to 5.99)

## 4. Further Research

After the systematic review, we conducted herb frequency statistics from the included 6 RCT articles and 10 patents for CSP. The result showed that 60 kinds of herbs were mentioned in 16 articles; 43 of them appeared once, 8 of them appeared 2–4 times, 4 of them were valued at 5–7 in frequency and 4 (*Lonicera japonica* Thunb, *Ophiopogon japonicus* Ker Gawl, *Glycyrrhiza uralensis* Fisch, and *Platycodon grandiflorum* A. DC.) were valued equal to or greater than 8. The details are shown in the following table (Table 2).

After looking up the relevant literature, we found that these four Chinese herbs have multiple effects; to make the article more specific, we decided to start with the etiology of CSP. We have qualitatively summarized the phytochemical investigations, pharmacological activities, and cytotoxic activities of the top four herbs in the form as follows.

## 5. Phytochemical Investigations

### 5.1. *Lonicera japonica*

*Lonicera japonica* contains numerous kinds of chemical components, mainly including flavonoids, organic acids, volatile oils, iridoids, and saponins. The chlorogenic acid and flavonoid luteolin of organic acids are recognized as essential indicators, especially for the quality inspection of many Chinese medicine preparations [20].

#### 5.1.1. Organic Acids

*Lonicera japonica* contains a variety of organic acids, such as ferulic acid, protocatechuic acid, and chlorogenic acid. [21]; the primary effective components are chlorogenic acid compounds, including chlorogenic acid, isochlorogenic acid, caffeic acid, 3,5-dicaffeoylquinic acid and so on, in which caffeic acid has the strongest activity [22].

#### 5.1.2. Flavonoids

The flavonoids in *Lonicera japonica* were first reported by Gao et al. [23] in 1995, including flavonoids and flavonoids. The isolated flavonoids [24] refers to luteolin, luteolin-7-O-*β*-D-glucoside, 5-hydroxy-7, 4′dimethoxy flavones, luteolin-7-o-A-D-gluconicanhydride, quercetin-3-o-*β*-D, and hyperoxide. In 2017, Ni et al.'s study pointed out that quercetin-3-o-*α*-L-pyranorablycoside was first isolated from *Lonicera japonica* [25].

#### 5.1.3. Triterpenoid Saponins

Triterpenoids and triterpenoid saponins are active ingredients in *Lonicera japonica*. At present, more than 30 kinds of triterpenoid saponins have been found in it [26].

### 5.2. *Ophiopogon japonicus*

At present, various chemical components such as steroidal saponins, high isoflavones, and polysaccharides have been isolated from different parts of *Ophiopogon japonicus* [27]. Among them, steroidal saponins and high isoflavones are considered to be the main active ingredients since they have multiple pharmacological activities [28].

#### 5.2.1. Steroidal Saponins

Reports showed that 64 kinds of steroidal saponins had been found in *Ophiopogon japonicus* (L.f) Ker Gawl and Liriope Lour, the saponins with ruscogenin and diosgenin account for a large proportion. Currently, ruscogenin and ophiopogonin A, B, B′, C, C′, D, and D′ have been studied in depth, and in fact, the aglycon of Ophiopogonin B′, C, and D′ is diosgenin, while the aglycon of ophiopogonin A, B, C, and D is ruscogenin [29].

#### 5.2.2. Homoisoflavonoids

Homoisoflavonoids are also major components of *Ophiopogon japonicus*, and more than 30 kinds of homoisoflavone compounds have been isolated from the herb [30–38].

### 5.3. *Glycyrrhiza uralensis*

*Glycyrrhiza uralensis* contains hundreds of compounds such as glycyrrhizic acid, gany glycyrrhizin, glycyrrhizin, 7-methylcoumarin, and umbelliferone. Flavonoids, saponins, and polysaccharides are the most important physiological active ingredients of *Glycyrrhiza uralensis* [39].

#### 5.3.1. Saponins

Triterpenoid saponins are iconic components in *Glycyrrhiza uralensis*, chiefly found in the rhizome and root of *Glycyrrhiza uralensis*. They are early recognized and developed for higher content, and the sweetness is 10 times higher than sucrose. Heretofore, dozens of saponins, principally in the form of glucose glycosides with good water solubility, have been isolated from three common *Glycyrrhiza uralensis*, such as Ural *Glycyrrhiza uralensis*, Radix Glycyrrhizae Glabrae, and Glycyrrhiza inflata Batalin [40].

#### 5.3.2. Flavonoids

The flavonoids in *Glycyrrhiza uralensis*, including glycyrrhizin, isoglycyrrhizin, and neoisoglycoside, have significant medicinal functions such as antiulcer, antibacterial, anti-inflammatory, and hypolipidemic. With the addition of its moisturized effect, *Glycyrrhiza uralensis* has been used as an important raw material of anti-inflammatory and antiallergic agents for high-end skin care products [41]. Until now, more than 130 kinds of flavonoids [42] have been isolated from the aerial parts, rhizomes, and roots of *Glycyrrhiza uralensis*, more than 30 kinds of which are isoflavonoids [43].

#### 5.3.3. Polysaccharide Components

In 1954, the literature recorded the separation and identification of more than 10 kinds of polysaccharides from *Glycyrrhiza uralensis* [44], and now new polysaccharides have been isolated. Zhou et al. [45] with the use of high performance capillary electrophoresis, indicated that *Glycyrrhiza uralensis* polysaccharides were composed of rhamnose, dextran (as the backbone), arabinose, and galactose. Glycyrrhiza polysaccharide has significant antiviral, antioxidant, and immune-enhancing effects.

### 5.4. *Platycodon grandiflorum*

The chemical components contained in *Platycodon grandiflorum* include triterpenoid saponins, polysaccharides, flavonoids, sterols, fatty acids, and trace elements.

#### 5.4.1. Triterpenoid Saponins

Triterpenoid saponins are the first components that have been isolated from *Platycodon grandiflorum* and reported [46]. At present, more than 70 kinds of triterpenoid saponins have been isolated and identified [47, 48]. They were classified into platycodin, polyglactin, platycogenic acid, platycodin lactone, and other atypical triterpenoid saponins in Fu et al. [49, 50] according to the type of aglycon.

#### 5.4.2. Flavonoids

Flavonoids mainly exist in the aerial part of *Platycodon grandiflorum*. Piao et al. [51] using DPPH and ABTS methods, showed that the order of the flavonoid glycoside content in different parts from high to low was leaves, stem, and root, flavonoid glycosides was hardly detected in the root. Up to now, 11 kinds of flavonoids have been isolated and identified [47]. According to research, flavonoids have anti-inflammation, antibacteria, anti-tumor, and other biological activities [48].

## 6. Pharmacological Activities

### 6.1. Anti-Influenza Virus Effect

#### 6.1.1. *Lonicera japonica*

Influenza virus (Orthomyxoviridae) is a phased negative-stranded RNA virus that can cause human and poultry infections. The influenza A virus, recognized as the most harmful one, mutates readily and has consequently caused several pandemics.

The main active ingredients for anti-influenza virus in *Lonicera japonica* are chlorogenic acid, caffeic acid, quercetin, hibisin, and so on [52–55]. Modern pharmacological studies showed that the pharmacological actions and its mechanisms of *Lonicera japonica* against influenza virus primarily included the inhibition of viral proliferation, protection of lung injury, and regulation of immunity. The active constituents of *Lonicera japonica* could inhibit viral activities, reduce the release of inflammatory factors, and promote lymphocyte proliferation in the body [52, 56]. In addition, miR2911 in *Lonicera japonica* is expected to be a breakthrough of anti-influenza virus gene drugs due to its unique structure and activity [57].

#### 6.1.2. *Glycyrrhiza uralensis*

Utsunomiya et al. [58], used the mouse model to study the anti-influenza virus effect of glycyrrhizin, turning out that vaccinated mice survived 100% while others were infected and died. However, the inhibition of influenza virus has not been found *in vitro* experiments. It is believed that the mechanism of action is related to the creation of interferon-gamma by T-lymphocytes induced by glycyrrhizin.

### 6.2. Anti-Respiratory Syncytial Virus

#### 6.2.1. *Lonicera japonica*

Studies showed that *Lonicera japonica* had a direct inactivation effect of RSV in Hela cells [59], and the inactivation was enhanced while the concentration was increasing, when IC 50 was 45 mg/mL and TI was 31.2. The replication and proliferation of the virus were also significantly inhibited by *Lonicera japonica*, and the effectiveness was superior to the control group with ribavirin, when IC 50 was 30 mg/mL and TI was 10.5. It had a certain inhibitory effect on the adsorption phase of the virus when IC 50 is 1 mg/mL and TI is 5.0, yet had no blocking effect on the invasion of cells by RSV virus.

#### 6.2.2. *Glycyrrhiza uralensis*

*Glycyrrhiza uralensis* and some of its active ingredients have the antibacterial effect *in vitro* on various pathogenic bacteria of the respiratory tract, such as *staphylococcus*, *streptococcus*, *Haemophilus influenzae*, *Bacillus subtilis*, Proteus, *Klebsiella pneumoniae*, *Legionella* and *Moraxella catarrhalis* [60]. *Glycyrrhiza uralensis* has been proved to be effective in inhibiting the human respiratory syncytial virus (HRSV), but it was unclear whether *Glycyrrhiza uralensis* or *Glycyrrhiza uralensis* preparata would be better and which constituent was active until Feng et al. [61]. They did thrombocytopenic experiments with HEp-2 and A549 cell lines and detected the active constituents for the antirespiratory syncytial virus in the hot water extracts of *Glycyrrhiza uralensis* formulas, such as glycyrrhizic acid and 18*β*-glycyrrhetic acid (18*β*-GA). The abilities of crude *Glycyrrhiza uralensis* to inhibit viral replication and to stimulate IFN-*β* were evaluated by reverse transcription polymerase chain reaction (RT-PCR) and enzyme-linked immunosorbent assay (ELISA), respectively. The results showed that *Glycyrrhiza uralensis* had better effects on HEp-2 cells than *Glycyrrhiza uralensis* preparata, and there was no difference on A549 cells; glycyrrhizin was ineffective, and 18*β*-GA exhibited potent anti-HRSV activity. Therefore, they believed that both *Glycyrrhiza uralensis* and *Glycyrrhiza uralensis* preparata could effectively fight against HRSV infection of the upper respiratory tract epithelial cells; *Glycyrrhiza uralensis* could repress viral adhesion and internalization and inhibit HRSV by stimulating the secretion of interferons. 18*β*-GA might be one of its active constituents.

### 6.3. Antibacterial Effect

#### 6.3.1. *Lonicera japonica*

Traditional Chinese herbs are the essence of nature; many of them are anti-infectives and rarely result in drug resistance, which can provide new ideas for antibacterial [62]. *Lonicera japonica* has a certain inhibitory effect on a variety of bacteria and is a typical broad-spectrum antibacterial Chinese medicine.

*(1) Effect on Gram-Positive Bacteria*. The research on anti-Gram-positive bacteria by *Lonicera japonica* principally focused on *Staphylococcus aureus*, *Streptococcus pneumoniae*, *Staphylococcus epidermidis*, *Streptococcus hemolyticus*, *Enterococcus,* and *Bacillus cereus* [62]. Huang et al. [63] studied the antibacterial activity of compound *Lonicera japonica* external lotion and *Lonicera japonica* water extracts and found that they both had antibacterial actions against *Staphylococcus aureus*, *Escherichia coli*, *Bacillus subtilis,* and *Pseudomonas aeruginosa*. Chan et al. [64] found that *Lonicera japonica* aqueous extracts (MIC ranged from 37.5 to 100 mg/mL) had a strong antibacterial effect against *Staphylococcus aureus* standard strain, *Staphylococcus anhemolyticus*, *Streptococcus pneumoniae*, *Enterococcus faecalis*, *Bacillus pumilus*, *Bacillus subtilis*, *Staphylococcus epidermidis*, *Streptococcus hemolytic*-*β,* and *Staphylococcus cohnii*. Ruan et al. [65] reported that the water extracts of *Lonicera japonica* had a significant antibacterial effect against *Staphylococcus aureus* and *Streptococcus*; its alcohol extracts and water extracts had certain antibacterial ability against *Streptococcus suis* type II.

*(2) Effect on Gram-Negative Bacteria*. The experimental strains of Gram-negative bacteria are mostly Enterobacteriaceae bacteria, including *Escherichia coli*, *Klebsiella pneumoniae*, *Salmonella, Shigella*, and nonfermenting bacteria *Pseudomonas aeruginosa* and *Acinetobacter baumannii*. Generally, *Lonicera japonica*'s antibacterial effect on Gram-negative bacteria is lightly inferior to that on Gram-positive bacteria.

Studies showed that [66, 67] *Lonicera japonica* extracts and nanosilver particles had broad application prospects with their strong inhibitory effect on *Escherichia coli*; Mu et al. [68], reported that *Lonicera japonica* extracts had certain antibacterial activities on clinically isolated pneumonia Kreb bacteria. They also claimed that using Chinese herbal medicine for bacterial infectious diseases could prevent drug resistance quickly developed by bacteria and reduce side effects caused by excessive use of antibacterial drugs.

Chlorogenic acid, the main component of *Lonicera japonica*, has a significant effect on inhibiting the growth of *Meningococcus*, *Salmonella typhi*, and *Escherichia coli*. The antibacterial mechanism is related to the noncompetitive inhibition of arylamine-*N*-acetyltransferase (NAT) in bacteria [69]. By detecting the conductivity, alkaline phosphatase activity, absorbance, and penetration of the hydrophobic fluorescent dye 1-*N*-phenylnaphthylamine (NPN) on the bacterial membrane and the wall, it was found that chlorogenic acid could increase the permeability of the cell wall of *Escherichia coli*, allowing cell electrolytes, enzymes, etc. to penetrate into the membrane and the wall, consequently leading to apoptosis [70].

#### 6.3.2. *Ophiopogon japonicus*

Liang et al. [71] detected the antibacterial effect and inhibition of peptide deformylase (PDF) when using the fluorescence method and MTT method to the endophytic fungi of *Ophiopogon japonicus*.

#### 6.3.3. *Glycyrrhiza uralensis*

Modern experimental studies showed that the water extracts, methanol extracts, and supercritical extracts of *Glycyrrhiza uralensis* had considerable inhibitory effects on variety of Gram-negative and Gram-positive bacteria [72]. Wang et al. [73] found that glycyrrhizic acid could significantly increase the production of reactive oxygen species and nitrogen, promote the expression of antibacterial genes, activate chicken macrophages, and enhance the ability of phagocytic cells and *Salmonella.*

#### 6.3.4. Platycodon grandiflorum

It was found that platycodin D had antibacterial activity and, with the increase of concentration of platycodin D, could gradually reduce the adhesion and viability of candidosis of *C. albicans*, inhibit its transformation from spore to mycelial phase, decrease the content of IL-8 and HBD-2 protein in supernatant, and the expression of HBD-2 mRNA in KB cells. The study indicated that platycodin D might reduce the infection of oral mucosa of *Candida albicans* by its immunosuppressive effect on oral mucosal epithelial cells [74].

### 6.4. Antipyretic and Anti-Inflammatory Effects

#### 6.4.1. *Lonicera japonica*

Ryu et al. [75], pointed out that *Lonicera japonica* extracts had good anti-inflammatory and analgesic effects and performed better when absorbed by intestine. *Lonicera japonica* could also be used in the treatment of pertussis, pneumonia, acute appendicitis, acute mastitis, influenza, and other diseases. Some scholars used cigarette smoke extracts to stimulate KB cells, constructed an acute oral inflammation model and studied the antiacute oral inflammation of *Lonicera japonica* [76]. It was found that the *Lonicera japonica* could inhibit the expression of TNF-α, IL-6, and IL-8 and promote the decrease of IL-10 secretion, which was dose-dependent, suggesting that *Lonicera japonica* had a certain therapeutic effect on oral inflammation. The flavonoids of *Lonicera japonica* could also inhibit the inflammatory mediators TNF-*α* and IL-1*β* through the PI3K/Akt/NF-kB signaling pathway [77]. The extracts of *Lonicera japonica* and chlorogenic acid acted as an activator of the AMPK/Nrf2 pathway and could inhibit phosphorylation of NF-kB, JAK-1, STAT-1, and MAPK pathways, thereby reducing acute and chronic inflammatory responses induced by lipopolysaccharides [78].

The luteolin derived from *Lonicera japonica*, possible by the inhibition of ERK1/2, JNK1/2, and NF-kB pathway, could regulate TNF-*α*, IL-6, IL-8, and GM-CSF, decrease COX-2 expression, and intracellular Ca 2 release [79].

#### 6.4.2. *Ophiopogon japonicus*

*Ophiopogon japonicus* could reduce the production of nitric oxide and proinflammatory cytokines by inhibiting the phosphorylation of ERK1/2 and JNK in the MAPK signaling pathway and exerting significant anti-inflammatory activity [80].

Total saponins of *Ophiopogon japonicus* could inhibit endothelial cell apoptosis and upregulate the expression of the endothelial cell adhesion factor (CD31) to play anti-inflammatory effect [81].

Tian et al. [82] studied the in vivo anti-inflammatory activity of water extracts (Lm-a), total saponins (Lm-s), and main components (Lm-3) of *Ophiopogon japonicus* by establishing the inflammatory model of mice auricle-swelling and carrageenan- or histamine-induced swelling of the ankle of mice, and examined the *in vitro* anti-inflammatory activity of Lm-3 by tumor necrosis factor-*α* (TNF-*α*) or phorbol ester (PMA)-induced myeloid leukemia cell line (HL-60) and the human umbilical vein endothelial cell (ECV304) adhesion model. It was found that Lm-a, Lm-s, and Lm-3 could significantly alleviate p-xylylene-induced ear swelling and carrageenan-induced paw swelling, and the mechanism might be related to the regulation of proteinkinase C pathway.

Ma et al. [83] also found in vitro that ruscogenin could strikingly inhibit the adhesion of cytokine TNF-*α*-induced HL-60 cells to ECV304 cells.

#### 6.4.3. *Glycyrrhiza uralensis*

The active ingredient of *Glycyrrhiza uralensis* has a good anti-inflammatory effect. Studies showed that the total saponins from wild *Glycyrrhiza uralensis* in Gansu could inhibit the secretion of nitric oxide (NO), tumor necrosis factor alpha (TNF-*α*), and interleukin (IL)-6 inflammatory factors to achieve anti-inflammatory effects [84].

Li et al. [85] found that *Glycyrrhiza uralensis* total saponins could not only significantly reduce the release of NO, IL-1, and TNF-α from lipopolysaccharide (LPS)-induced macrophage cell line RAW264.7, but also reduce the synthesis of the arachidonic acid metabolic pathway PGE 2 by inhibiting the phospholipase A2 (PLA 2) enzyme activity and cyclooxygenase-2 (COX-2) expression. Matsui et al. [86] studied on the structure-activity relationship of *Glycyrrhiza uralensis* and found that glycyrrhizin itself had direct anti-inflammatory effects in vitro, not depending on glucocorticoids.

Xie et al. [87] used the airway instillation (LPS)-induced mouse pneumonia model to study the anti-inflammatory mechanism of flavonoids in *Glycyrrhiza uralensis*. The results showed that total flavonoids of *Glycyrrhiza uralensis* reduced neutrophils recruitment by inhibiting inflammatory cell infiltration and the release of mediators and therefore relieved the oxidative damage of neutrophils.

#### 6.4.4. *Platycodon grandiflorum*

The anti-inflammatory activity of *Platycodon grandiflorum* is mainly due to the presence of platycodin, which has the strongest anti-inflammatory activity of platycodin D and D3 and the inflammatory model induced by phorbol ester (TPA), which inhibits the production of prostaglandin E2 [88]. For the model of inflammation induced by lipopolysaccharide and platycodin D and D3 could inhibit NO production and increase TNF-α secretion [89].

Studies showed that platycodon could not only effectively improve the symptoms of bronchial asthma, but also prevent bronchitis; high dose group of *Platycodon grandiflorum* could effectively prolong the asthmatic latency of asthmatic guinea pigs, significantly inhibit the generation and release of oxygen-free radicals, effectively promote asthma guinea pig *γ*—interferon (IFN-*γ*) and human lipoxin A4 (LXA4) release, indirectly balance helper T cells (Th) 1/Th2, and regulate LXA4 in the body to exert anti-inflammatory and prodissipative effects [90]. Platycodin saponins lowered the level of granulocyte-macrophage aggregation stimulating factor (GM-CSF), reduced the expression of interleukin (IL-13) in cells, and inhibited the activation of NF-kb and the MAPK pathway to achieve anti-inflammation [91]. Platycodin saponins could fight with various inflammations by inhibiting the PGE2 (prostate) pathway and NO (nitric oxide) secretion.

In addition, total saponins of *Platycodon grandiflorum*, in the antiallergic test and the skin test of mast cells *in vitro*, had significant inhibition of the release of hexosaminidase and histamine and were potential to be developed as antiallergic drugs [92].

### 6.5. The Role of Protecting the Lungs

#### 6.5.1. *Lonicera japonica*

In an experiment evaluating the biological effects of *Lonicera japonica* on a mouse model of chronic obstructive pulmonary disease, inhalation of *Lonicera japonica* microparticles reduced the levels of TNF-*α* and IL-6 in bronchoalveolar fluid of mice with chronic obstructive pulmonary disease and allowed them to be in peripheral blood. The number of inflammatory cells including neutrophils is reduced. In addition, *Lonicera japonica* microparticles can induce the recovery of elastin and collagen distribution in the lung tissue of mice with chronic obstructive pulmonary disease, and reduce the expression of Caspase-3. The above experimental results show that inhaled *Lonicera japonica* particles have a good development prospect in the treatment of chronic obstructive pulmonary disease [93].

#### 6.5.2. *Ophiopogon japonicus*

Ruscogenin has a remission effect on lung injury [94]. It was observed on sacrificed ICR mice, after one-hour oral administration of ruscogenin (0.3, 1.0, and 3.0 mg·kg^−1^) followed with eight-hour induction of lung injury by lipopolysaccharide (LPS) 30 mg·kg^−1^ (iv), which ruscogenin significantly reduced the ratio of wet weight/dry weight of the lungs and improved various histopathological changes (pulmonary edema, inflammatory cell aggregation and infiltration, etc.), which suggested that ruscogenin might inhibit the tissue factor, induce the nitric oxide synthase expression, and improve the activation of NF‐kB p65 to relieve the lung injury induced by LPS [95].

#### 6.5.3. *Glycyrrhiza uralensis*

Qamar et al. [96] investigated the protective effects of glycyrrhizic acid (GA) on benzo(a)pyrene-induced lung injury in Wistar rats. Glycyrrhizic acid was administered orally to Wistar rats at a dose of 50 mg/kg and 100 mg/kg. The results showed that glycyrrhizic acid reduced the total number of proteins, total cells, and elasticity by inhibiting the activity of caspase in the lung tissue in lung epithelial cells. Protease activity enhanced LDH and ALP activity on phospholipids in BALF for protection. Zhang et al. [97] found that *Glycyrrhiza uralensis* flavonoids could inhibit the synthesis of proinflammatory cytokines TNF-*α*, IL-6, and IL-1*β* induced by lipopolysaccharide (LPS). HE staining of the lung tissue could directly prove that *Glycyrrhiza uralensis* flavonoids had protective effects on acute lung injury and could improve histological changes such as inflammatory cell infiltration and interstitial edema in the lungs.

#### 6.5.4. *Platycodon grandiflorum*

Liu et al. [98] found that platycodin D could effectively reduce the content of CI, PIIIP, and HA in rat serum, down-regulate the expression of TGF-*β*m RNA in rat lung tissue, and effectively improve the general clinical symptoms of pulmonary fibrosis induced by bleomycin hydrochloride in rats. It was speculated that it might inhibit the synthesis of extracellular matrix in the lung tissue of mice with pulmonary fibrosis and promote its degradation, thereby reducing collagen deposition and inhibition during the formation of pulmonary fibrosis. The expression of TGF-*β*m RNA in rat lung tissue reached the antifibrotic effect.

Chen et al. [99] found that platycodin saponins could significantly alleviate the pathological changes of airway remodeling in chronically branched mice. The intervention mechanism might be through the removal of inflammatory factors and free radicals in the lung tissue of chronically icy mice [100]. At the same time, it could inhibit the expression of MMP9 and TIMP-1, so as to reduce the deposition of collagen to reduce tracheal stenosis and achieve the purpose of reversing airway remodeling.

Yao et al. [98] reported that total saponins of *Platycodon grandiflorum* might significantly reduce the inflammatory pathology of the lung tissue induced by PM2.5 by regulating the balance of inflammatory cytokines, and inhibiting the process of pulmonary fibrosis by reducing the expression of TGF-*β* gene and protein to protect and repair the lung tissue.

### 6.6. Antitussive Effect

#### 6.6.1. *Ophiopogon japonicus*

Studies showed that the Chinese herbal formula “Maimendong Decoction” had an antitussive effect and was now used to treat dry cough caused by pharyngitis and bronchitis [101]. Studies have also confirmed that in the animal model of bronchitis, Maimendong Decoction could inhibit the increase of tracheal vagal afferent nerve electrical stimulation, indicating that the site of antitussive effect of Maimendong Decoction may be in the trachea [102].

Some foreign scholars have found that the pharmacological effects of Maimendong Decoction on antitussiveness were consistent with OP-D, indicating that OP-D may be one of the antitussive ingredients in Maimendong Decoction, and the nystatin perforated membrane was used. The clamp technique was used to investigate the effect of OP-D on the parasympathetic nerves of the acutely isolated rat paratracheal ganglia. It was found that OP-D exerts hyperpolarization on paratracheal neurons by activating *K* channels, which clarified the mechanism of action of OP-D antitussive [101] to some extent. Tang et al. [103] studied the antiasthmatic and antiallergic effects of Ophiopogon japonicus polysaccharide (POT) through animal experiments. The results showed that Ophiopogon japonicus polysaccharide could inhibit the contraction of bronchial smooth muscle, reduce the occurrence of asthma, and had a more significant effect on mouse skin allergies; in addition, the saponin A and the saponin B could inhibit the expression of eosinophilic chemokines induced by IL-4 and TNF-*α* [104], and the prospect of these three compounds was brilliant in the treatment of allergic bronchial asthma.

#### 6.6.2. *Glycyrrhiza uralensis*

*Glycyrrhiza uralensis* has been a good medicine for antitussive and cough since ancient times. The preparation of *Glycyrrhiza uralensis*, such as compound *Glycyrrhiza uralensis* tablets, is also a common drug for the treatment of cough and asthma in clinical practice. Han et al. [105] found that the content of glycyrrhetinic acid in the water extracts of *Glycyrrhiza uralensis* after transformation by intestinal flora increased significantly, and the inhibition rate of cough latency and cough frequency of experimental mice was higher than that before transformation.

Yu et al. [106] found that the geraniol extract, *Glycyrrhiza uralensis* flavonoids, and glycyrrhetinic acid could obviously relieve the cough and eliminate phlegm on mice with ammonia cough and SO_2_ cough test. Wu [107] found by intravenous injection of sodium glycyrrhetinate that it had antitussive and expectorant effects on the mouse cough model caused by ammonia spray, and its antitussive effect was weaker than codeine but not addictive.

#### 6.6.3*. Platycodon grandiflorum*

*Platycodon grandiflorum* has excellent antitussive and expectorant activity. According to statistics, only the Chinese pharmacopoeia contains 65 kinds of antitussive and expectorant formulas containing platycodon. Zhu et al. [108] selected 9 different origins of *Platycodon grandiflorum* and found that it has significant antitussive and antitussive effects, and the effects of different producing areas are obviously different.

In the Chinese Pharmacopoeia 2005 edition, the total saponin content of *Platycodon grandiflorum* is not less than 6%; in the Chinese Pharmacopoeia 2015 edition, the content of platycodin D (C_57_H_92_O_28_) is not less than 0.10%. However, studies have shown that the cough and sputum activity of *Platycodon grandiflorum* has no obvious correlation with the content of platycodin D, and it has no obvious correlation with the total saponin content of *Platycodon grandiflorum*, suggesting that its activity has a complex relationship. Therefore, further research is needed to clarify the memory correlation between antitussive and antispasmodic activities and components [108, 109].

## 7. Cytotoxic Activity

### 7.1. *Lonicera japonica*

The tetraploid was tested for acute toxicity by diploid *Lonicera japonica* medicinal materials [110]. The pretest was used to determine the median lethal dose. The mice with tetraploid and diploid *Lonicera japonica* were calculated by the BLISS method. The median lethal dose (LD50) was 72.12 g/kg and 69.92 g/kg, respectively, which were equivalent to 412 times and 400 times of clinical doses, indicating that tetraploid and diploid *Lonicera japonica* have lower acute toxicity and larger safety range. From the perspective of chemical composition of tetraploid *Lonicera japonica*, the quality of tetraploid *Lonicera japonica* medicinal materials is unstable and needs further study. From acute toxicity studies and related pharmacodynamic studies, tetraploid *Lonicera japonica* medicinal materials are acutely toxic and related drugs. There is no significant difference between diploid and *Lonicera japonica*, and it is safe and effective in clinical application.

However, *Lonicera japonica* is cold, and if it is taken too much, it will cause adverse reactions. Pu et al. studied the toxic effects of cold Chinese medicine on mice and found that the content of copper, zinc, magnesium, and phosphorus in the liver tissue of mice was significantly increased after drinking *Lonicera japonica*. The manganese content increased slightly, the iron content decreased significantly, the color of the liver became darker, the morphology did not change significantly, the spleen became smaller, the body hair was dark yellow, the body shape became thinner, and the activity decreased. Compared with the control group, the difference was not statistically significant [111].

### 7.2. *Ophiopogon japonicus*

Lin et al. [112] studied the cytotoxicity of dextran D′ on rat cardiomyocyte H9c2 and found that higher concentration of dextran D's had a significant cytotoxic effect on H9c2 cardiomyocytes. Hu et al. [113], used the mouse lymphoma cell test (MLA) and mouse bone marrow micronucleus test (MNT) to study the genotoxicity of Ophiopogon japonicus decoction and found that Ophiopogon japonicus decoction in ±S9 conditions (nonmetabolic activation (−S9) and metabolic activation (+S9) conditions could induce tk locus mutation in L5178Y cells, suggesting that there might be mutagenic substances; however, the test article had no damage to mouse bone marrow cells and was activated by metabolism *in vivo*. Genotoxic effects were not shown. In addition, the aqueous extract of *Ophiopogon japonicus* had no significant effect on maternal and embryo/fetal development of SD rats. There was no obvious maternal toxicity and embryonic/fetal developmental toxicity in wheat winter water extract [114].

### 7.3. *Glycyrrhiza uralensis*

In order to evaluate the toxicological safety of the aerial part of the *Glycyrrhiza uralensis*, Zhao et al. [115] used the acute oral toxicity test, the mouse bone marrow micronucleus test, the mouse sperm abnormality test, and the safety test method based on the inflammatory model. It turned out that the maximum tolerated dose (MTD) of water extraction on the aerial part of *Glycyrrhiza uralensis* to the mice was 96 g·kg^−1^, but it was not shown to be genotoxic to mice. Its safety still needs further study.

As a traditional Chinese medicine, *Glycyrrhiza uralensis* is widely used in the prescription of traditional Chinese medicine. Therefore, *Glycyrrhiza uralensis* has the name of “ten-party and nine-grass.” The mechanism of toxicity and attenuation of *Glycyrrhiza uralensis* after compatibility with toxic Chinese medicine has many aspects. The mechanism of attenuating compatibility should be utilized, and the mechanism of toxicity and compatibility should also be paid attention to. Chen [116] found that glycyrrhizic acid and Ganzi could form molecular complexes with Ganzi during decoction, increasing the dissolution rate of toxic substances of Ganzi and increasing the toxic components of decoction. Jin et al. [117] used intraperitoneal injection of scutellaria or seaweed decoction and subcutaneous injection of *Glycyrrhiza uralensis* decoction and found that the toxicity of sassafras increased with the increase of *Glycyrrhiza uralensis* dosage. Studies suggested that the increase in toxicity was not due to changes in the physical and chemical properties of the mixture, but might be the result of interactions between the drug and the animal. The study also found that Ganzi might increase the expression and activity of CYP2E1 in rats and promote the conversion of precarcinogens and protoxins into carcinogens and poisons, leading to toxic effects on the body, and then confirmed that, at the level of mRNA, protein expression and enzyme activity, there might be drug-drug interactions based on the mechanism of drug metabolism enzymes in Ganzi and *Glycyrrhiza uralensis* [118].

### 7.4. *Platycodon grandiflorum*

In order to study whether the extract of Platycodon grandiflorum as a feed additive and long-term application in the livestock and poultry industry will cause toxic reactions, the toxicological test of the water extract of *Platycodon grandiflorum* was carried out [119]: In the acute toxicity test, when LD50 > 30360 mg/kg, there were no deaths or other abnormal behaviors in the mice, indicating that the water extract of the platycodon is an actual nontoxic substance. In the accumulation toxicity test, during the observation period and during the 7-day withdrawal period, there was no death in the mice of each test group and no abnormality in spirit, diet, and drinking water, and no other abnormal behavior occurred. After 20 days of administration, there was no abnormality and death in the mice of each test group and control group. At the same time, the average body weight gained, and the average material consumption and organ coefficient of the mice in the experimental group were not significantly different from those of the control group. The difference indicated that the water extract of *Platycodon grandiflorum* had no accumulation effect. It proved that the water extract of *Platycodon grandiflorum* was nontoxic and has a better application prospect than antibiotics.

## 8. Discussion

As was presented above, the water extracts of *Lonicera japonica*, *Ophiopogon japonicus*, *Glycyrrhiza uralensis*, and *Platycodon grandiflorum* all assuredly have certain antibacterial, antipyretic, and anti-inflammatory effects; all these herbs have the effect of protecting the lungs except for *Lonicera japonica*. CSP is chiefly caused by pathogenic microbial infection, allergic reaction, and inflammation adjacent to the lungs, which indicates that the aforementioned four Chinese herbs are in line with the principle of symptomatic treatment. Additionally, in the theory of traditional Chinese medicine, the throat is internally related to the lung, which is the organ that controls the respiration and immunity; therefore, the health of the throat depends on the lung to some extent. In the clinical, the treatment of CSP with herbal tea has been promoted currently and the systematic evaluation showed a good clinical efficacy.

According to the statistical results, the frequencies of *Lonicera japonica*, *Ophiopogon japonicus*, *Glycyrrhiza uralensis,* and *Platycodon grandiflorum* are significantly higher than other traditional Chinese herbs, which are based simply on the statistics of related literature. Thus far, no efficacy evaluation of the herbal tea made with these four kinds of herbs has been reported, and the study on the interaction of active ingredients in water extracts of these four herbs is still waiting to be conducted. In our research, the Chinese herbal tea indeed contains ingredients that can alleviate CSP symptoms, but the current studies on herbal tea in the treatment of CSP are still at a relatively rough stage, obviously shown by the evaluation of literature quality. Systematic meta-analysis is difficult to be performed as the qualities of RCT articles are generally low.

## 9. Conclusion

In the theory of traditional Chinese medicine, the concept of “seven emotions” refers to seven methods in compatibility of ingredients, including using alone, mutual reinforcing, assisting, detoxifying, being detoxified, inhibiting, and antagonizing. Generally, the compatibility of two or more Chinese herbs does not simply give rise to an additive effect; the interactions are still in the study when it comes to traditional Chinese medicine compounds. Moreover, traditional Chinese medicine believes that syndromes can vary considerably with the cause, which literally means different prescriptions for different syndromes; thus, more attention should be paid in the clinical diagnosis and treatment of CSP.

In the field of Chinese herbal botany, further studies on the components of the water extracts of those four kinds of herbs are expected because the active constituents would be the closest way to understanding herbal tea. Besides, most of the toxicological studies on those four kinds of herbs were focused on animal experiments. Although testing on human body is, to some extent, unethical, yet the abnormal performance of medicine on mice is still different from that on human beings. The gap needs to be discussed.

In clinic, the overall quality of clinical research on Chinese medicine still needs to be improved, and the various elements of randomized controlled trials have to be standardized so that reliable articles could be obtained to accurately evaluate the efficacy of Chinese herbal tea.

Herbal tea in the treatment of CSP has the characteristics of convenience, economy, relative safety, and certainly effective. It is indeed worth popularization and further study in clinic.

## Figures and Tables

**Table 1:** General characteristics of the included articles and score of the modified Jadad scale.

Study	*N* (*T*/*C*)	Gender (M/F)	Age (years)	Treatment measure	Jadad scale score
Zhao [14]	25/25	*T*: 12/13	*T*:25.54 ± 48.64	*C*: cefuroxime axetil; loquat dew; Yan Li Shuang	3
		*C*: 12/13	*C*:24.31 ± 46.51	Mouth containing dropping pills	
Wang [15]	100/100	125/75	20–50	*C*: gentamicin injection; dexamethasone injection; chymotrypsin injection; atomization inhalation	3
Zeng et al. [16]	67/59	51/75	21–72	*C*: cefprozi capsule; atomization inhalation; Yinhuang buccal tablet	3
Liu et al. [17]	45/41	55/31	18–60	*C*: Yinhuang buccal tablet	2
Zhang and Liu [18]	60/60	Unknown	18–65	C: Qingyan pill	3
Wang and Li [19]	52/16	Unknown	18–58	*C*: Cao Shan Hu Hanpian	2

**Table 2:** Frequency statistics of herbs appeared in six trials and ten patents.

Scientific name (with the author name)	Chinese name	Frequency
*Lonicera japonica* Thunb.	Jinyinhua	10
*Ophiopogon japonicus* Ker Gawl.	Mai dong	10
*Glycyrrhiza uralensis* Fisch.	Gancao	9
*Platycodon grandiflorum* A. DC.	Jie Geng	8
*Chrysanthemum indicum* L.	Yejuhua	6
*Stereulia lychnophora* Hance.	Pangda Hai	6
*Mentha haplocalyx* Briq.	Bohe	5
*Scrophularia ningpoensis* Hemsl.	Xuan Shen	5
*Lilium brownie* F. E. Brown var.	Baihe	4
*Lophatherum gracile* Brongn.	Danzhuye	4
*Citrus reticulata* Blanco.	Chenpi	4
*Mormordica grosvenorii* Swingle.	Luohanguo	3
*Canarium album* Raeusch.	Ganlan	3
*Prunus mume* Sieb.et Zucc.	Wumei	3
*Rehmannia glutinosa* Libosch.	Sheng Di Huang	2
*Scutellaria baicalensis* Georgi.	Huangqin	2

## References

[1] Hu Y. T. (2000). *Otolaryngology Book*.

[2] Kong W. J., Zhou L., Wang B. Q. (2015). *Otolaryngology Book*.

[3] Weber R. (2014). Pharyngitis. *Primary Care: Clinics in Office Practice*.

[4] Kumari J. O., Rajendran R. (2008). Effect of topical nasal steroid spray in the treatment of non-specific recurrent/chronic pharyngitis—a trial study. *Indian Journal of Otolaryngology and Head & Neck Surgery*.

[5] Kalra M. G., Higgins K. E., Perez E. D. (2016). Common questions about streptococcal pharyngitis. *American Family Physician*.

[6] Murray C. R., Chennupati K. S. (2012). Chronic streptococcal and non-streptococcal pharyngitis. *Infectious Disorders—Drug Targets*.

[7] Choby B. A. (2009). Diagnosis and treatment of streptococcal pharyngitis. *American Family Physician*.

[8] Zhang H., Zhang J., Zhang Q. J. (2012). Summary of misdiagnosed diseases of chronic pharyngitis. *Modern Chinese Doctor*.

[9] Zhao L. D., Zhang H. L., Li M. G. (2014). Treatment analysis of patients with chronic pharyngitis. *The World’s Latest Medical Information Digest*.

[10] Chai Y., Gao Z. Y. (2017). Not inferior to the decoction of the herbal tea. *TCM Health*.

[11] Liu L. T. (2017). Herbal tea in the Qing dynasty medical archives. *Chinese Journal of Integrated Traditional and Western Medicine*.

[12] Zhu J. N. (2017). *Preliminary Investigation on the Law of Medication Used in the Qing Dynasty on Herbal Tea Essence and the Modern Application of Herbal Tea*.

[13] Bian Q., An Y. (2016). Research progress of herbal tea in the treatment of chronic pharyngitis. *Xinjiang Traditional Chinese Medicine*.

[14] Zhao Y. H. (2017). Evaluation on the effect of Qingyan- herbal tea in the treatment of chronic pharyngitis. *Clinical Medical Literature E-Magazine*.

[15] Wang L. S. (2015). Clinical observation on 100 cases of chronic pharyngitis treated with herbal tea decoction. *Chinese Modern Pharmaceutical Applications*.

[16] Zeng G., Qu Z. L., Niu P. (2013). Clinical observation on 67 cases of chronic pharyngitis treated with self-made traditional Chinese medicine. *Chinese General Practice*.

[17] Liu C. M., Zhang L. X., Dong P. D. (2012). Clinical observation on treatment of chronic pharyngitis with Shuang-Yuan herbal tea. *Chinese Practical Medicine*.

[18] Zhang H. Q., Liu G. Y. (2011). Effect of Qingyin decoction on chronic pharyngitis in asthmatic patients with long-term inhalation of sulpiride. *Chinese Journal of Integrated Traditional and Western Medicine*.

[19] Wang B. L., Li Z. D. (1994). 120 cases of chronic pharyngitis treated with traditional Chinese herbal tea. *Journal of Changchun College of Traditional Chinese Medicine*.

[20] Ding X. Y., Lin Z. J., Wang D. (2019). Advances in studies on the components and pharmacological effects of *Lonicera japonica-forsythia*. *Shandong Science*.

[21] Song Y. L., Ni F. Y., Zhao Y. W. (2014). Advances in research on chemical constituents of *Lonicera japonica*. *Chinese Herbal Medicine*.

[22] Song Y. L., Wang H. M., Ni F. Y. (2015). Study on phenolic acids and their anti-inflammatory activities in *Lonicera japonica*. *Chinese Herbal Medicine*.

[23] Gao Y. M., Wang M. Z., Wang J. P. (1995). Study on chemical constituents of *Lonicera japonica*. *Chinese Herbal Medicine*.

[24] Liu Y. F., Li L. P., Zhu H. Y. (2018). Advances in research on chemical constituents and pharmacological action of *Lonicera japonica*. *Journal of Liaoning University (Natural Science Edition)*.

[25] Ni F. Y., Wen J. H., Li M. (2017). Study on chemical constituents of *Lonicera japonica*. *Chinese Herbal Medicine*.

[26] Wu J., Wang C., Yu H. C. (2019). Advances in studies on chemical constituents and pharmacological effects of *Lonicera japonica*. *Chinese Journal of Experimental Formulaology*.

[27] Sun X. Y., Yu F., Xiao W. (2018). Research progress on modern application of *Ophiopogon japonicus*. *Modern Chinese Medicine*.

[28] Peng W., Ma X., Wang J. (2018). Research progress on chemical constituents and pharmacological effects of *Ophiopogon japonicus*. *Chinese Herbal Medicine*.

[29] Ma H. B. (2013). *Study on Chemical Constituents of Ophiopogon Japonicus*.

[30] Guan J., Li S. P. (2010). Discrimination of polysaccharides from traditional Chinese medicines using saccharide mapping-enzymatic digestion followed by chromatographic analysis. *Journal of Pharmaceutical and Biomedical Analysis*.

[31] Tada A., Kasai R., Saitoh T., Shoji J. (1980). Studies on the constituents of *Ophiopogonis tuber*. V. isolation of a novel class of homoisoflavonoids and determination of their structures. 1. *Chemical & Pharmaceutical Bulletin*.

[32] Zhou C.-X., Zou L., Mo J.-X. (2013). Homoisoflavonoids from *Ophiopogon japonicus*. *Helvetica Chimica Acta*.

[33] Asano T., Murayama T., Hirai Y., Shoji J. (1993). Comparative studies on the constituents of *Ophiopogonis tuber* and its congeners. VII. studies on the homoisoflavonoids of the subterranean part of *Ophiopogon japonicus* Ker-Gawler cv. Nanus. 2. *Chemical & Pharmaceutical Bulletin*.

[34] Duan C.-L., Kang Z.-Y., Lin C.-R., Jiang Y., Liu J.-X., Tu P.-F. (2009). Two new homoisoflavonoids from the fibrous roots of *Ophiopogon japonicus* (Thunb.) Ker-Gawl. *Journal of Asian Natural Products Research*.

[35] Lan S., Yi F., Shuang L., Chen Jie W., Zheng X.-W. (2013). Chemical constituents from the fibrous root of *Ophiopogon japonicus*, and their effect on tube formation in human myocardial microvascular endothelial cells. *Fitoterapia*.

[36] Chang J.-M., Shen C.-C., Huang Y.-L. (2002). Five new homoisoflavonoids from the tuber of *Ophiopogon japonicus*. *Journal of Natural Products*.

[37] Watanabe Y., Sanada S., Ida Y., Shoji J. (1985). Comparative studies on the constituents of *Ophiopogonis tuber* and its congeners. IV. studies on the homoisoflavonoids of the subterranean part of *Ophiopogon ohwii okuyama* and *Ophiopogon jaburan* (Kunth) lodd. *Chemical & Pharmaceutical Bulletin*.

[38] Nguyen T. H., Van S. T., Porzel A., Franke K., Wessjohann L. A. (2003). Homoisoflavonoids from *Ophiopogon japonicus* Ker-Gawler. *Phytochemistry*.

[39] Li H. H., Qing M., Yu J. (2015). Research progress on *Glycyrrhiza uralensis*. *Journal of Inner Mongolia Medical University*.

[40] He W., Ning J., Wu J. J. (2016). Advances in studies on the interaction between chemical constituents of *Glycyrrhiza uralensis* and cytochrome P450. *Chinese Herbal Medicine*.

[41] Han Y. N., Cheng X. Y., Hou J. L. (2014). Process for purifying total flavonoids from the aboveground of *Glycyrrhiza uralensis* by macroporous resin. *Jilin Traditional Chinese Medicine*.

[42] Li W., Song X. B., Zhang L. J. (2012). Research progress on chemical constituents in *Glycyrrhiza uralensis*. *Journal of Liaoning University of Traditional Chinese Medicine*.

[43] Yong X. J. (2005). *Study on Extraction and Separation of Isoflavones and Chlorophyll from the Aboveground of Glycyrrhiza Uralensis*.

[44] Zhang Q., Ye M. (2009). Chemical analysis of the Chinese herbal medicine Gan-Cao (licorice). *Journal of Chromatography A*.

[45] Zhou R., Qi L., Wang Y. F. (1999). Isolation, purification and high-performance capillary electrophoresis analysis of *Glycyrrhiza uralensis*. *Analytical Chemistry*.

[46] Tada A., Kaneiwa Y., Shoji J., Shibata S. (1975). Studies on the saponins of the root of *Platycodon grandiflorum* A. De Candolle. I. isolation and the structure of platycodin-D. *Chemical & Pharmaceutical Bulletin*.

[47] Deng Y. L., Ren H. G., Ye X. W. (2019). Advances in research on the history, chemical composition and pharmacological effects of *Platycodon grandiflorum*. *Chinese Journal of Experimental Traditional Medical Formulae*.

[48] Xie X. X., Zhang C., Zeng J. X. (2018). Advances in studies on chemical constituents and pharmacological activities of Chinese medicine *Platycodon grandiflorum*. *Chinese Medicine Bulletin*.

[49] Fu W. W., Dou D. Q., Pei Y. H. (2006). Advances in studies on chemical constituents and biological activities of *Platycodon grandiflorum*. *Journal of Shenyang Pharmaceutical University*.

[50] Liu Q., Li W., Zheng Y. N. (2013). Advances in studies on triterpenoid saponins and pharmacological activities in *Platycodon grandiflorum*. *Journal of Jilin Agricultural University*.

[51] Piao X. M., Yu Y., Sin H. H. (2017). *In vitro* antioxidant activity and total polyphenols and flavonoid aglycone content of *Platycodon grandiflorum*. *Journal of Jilin Agricultural University*.

[52] Lu J. X., Liang R. X., Lin H. B. (2018). Advances in research on the role of *Lonicera japonica* against influenza virus. *Research and Practice on Chinese Medicines*.

[53] Hu K. J., Sun K. X., Wang J. L. (2001). *In vitro* antiviral effect of chlorogenic acid. *Journal of Harbin Medical University*.

[54] Sinisi V., Stevaert A., Berti F. (2016). Chlorogenic compounds from coffee beans exert activity against respiratory viruses. *Planta Medica*.

[55] Liu Z. K., Zhao J. P., Li W. C. (2016). Computational screen and experimental validation of anti-influenza effects of quercetin and chlorogenic acid from traditional Chinese medicine. *Scientific Reports*.

[56] Zhang M. L., Li F., Liu W. (2016). Advances in research on antiviral effect of Chinese medicine *Lonicera japonica*. *Journal of Liaoning University of Traditional Chinese Medicine*.

[57] Zhou Z., Li X., Liu J. (2015). Honeysuckle-encoded atypical microRNA2911 directly targets influenza A viruses. *Cell Research*.

[58] Utsunomiya T., Kobayashi M., Pollard R. B., Suzuki F. (1997). Glycyrrhizin, an active component of licorice roots, reduces morbidity and mortality of mice infected with lethal doses of influenza virus. *Antimicrobial Agents and Chemotherapy*.

[59] Zhang X., Zheng M. X., Zhu Z. B. (2014). Study on the anti-respiratory virus infection of *Lonicera japonica in vitro*. *New Chinese Medicine*.

[60] Zhang M. F., Shen Y. Q. (2009). Advances in studies on antibacterial and antiprotozoal pharmacology of *Glycyrrhiza uralensis*. *Journal of Clinical Drug Therapy*.

[61] Feng Y. C., Chih W. K., Chai C. L., Shieh D. E., Yen M. H., Chang J. S. (2013). Water extract of licorice had anti-viral activity against human respiratory syncytial virus in human respiratory tract cell lines. *Journal of Ethnopharmacology*.

[62] Li Y. W., Wang Z. S., Liu X. W. (2019). Advances in anti-infective effects of *Lonicera japonica*. *Chinese Modern Doctors*.

[63] Huang K. Y., Xie Z. X., Wu Q. H. (2017). *In vitro* transdermal test and antibacterial activity of compound *Lonicera japonica* external lotion and its single-flavored drug. *Modern Chinese Medicine*.

[64] Chan E. C., Tan S., Lim Y. (2011). Standardised herbal extract of chlorogenic acid from leaves of *Etlingera elatior* (Zingiberaceae). *Pharmacognosy Research*.

[65] Ruan Z. Y., Wang Z. M., Li J. J. (2017). Study on antibacterial activity of *Lonicera japonica* volatile oil and residue extract. *Modern Food Technology*.

[66] Yang L., Qu F., Aguilar Z. P., Wei H., Xu H., Xu H. (2016). Enhanced antimicrobial activity of silver nanoparticles-*Lonicera japonica* Thunb combo. *IET Nanobiotechnology*.

[67] Han J., Lv Q.-Y., Jin S.-Y. (2014). Comparison of anti-bacterial activity of three types of di-O-caffeoylquinic acids in *Lonicera japonica* flowers based on microcalorimetry. *Chinese Journal of Natural Medicines*.

[68] Mu T. J., Yang F. L., Jin H. H. (2015). Experimental study and clinical application of Chinese herbal medicines such as forsythia on the antibacterial activity of *Klebsiella pneumoniae*. *Western Journal of Traditional Chinese*.

[69] Wei P., Tan A. J., Lu S. M. (2017). Study on elimination of drug resistance of pathogenic *Escherichia coli* by traditional Chinese medicine. *Jiangsu Agricultural Sciences*.

[70] Zhou Z. E., Luo Q. S., Xiong J. H. (2014). Antibacterial mechanism of chlorogenic acid and isochlorogenic acid a against *Escherichia coli*. *Food Science and Technology*.

[71] Liang H., Xing Y., Cheng J., Zhang D., Guo S., Wang C. (2012). Antimicrobial activities of endophytic fungi isolated from *Ophiopogon japonicus* (liliaceae). *BMC Complementary and Alternative Medicine*.

[72] Nie X. H., Yin G. Y., Shi B. J. (2003). Antitumor activity and immunological activity of active components of *Glycyrrhiza uralensis* fisch *in vitro*. *Chinese Medicine*.

[73] Wang B.-K., Mao Y.-L., Gong L. (2018). Glycyrrhizic acid activates chicken macrophages and enhances their Salmonella-killing capacity *in vitro*. *Journal of Zhejiang University-Science B*.

[74] Zhu L. F., Wang B. (2017). Effect of platycodin D on oral mucosal epithelial cells infected with *Candida albicans*. *Chinese Journal of Pathophysiology*.

[75] Ryu K. H., Rhee H. I., Kim J. H. (2010). Anti-inflammatory and analgesic activities of SKLJI, a highly purified and injectable herbal extract of *Lonicera japonica*. *Bioscience, Biotechnology, and Biochemistry*.

[76] Li P. L., He L. L., Li C. Y. (2016). Comparison of anti-acute oral inflammation between *Lonicera japonica* and sinensis. *Journal of Sun Yat-Sen University (Natural Science Edition)*.

[77] Han M. H., Lee W. S., Nagappan A. (2016). Flavonoids isolated from flowers of *Lonicera japonica* Thunb. Inhibit inflammatory responses in BV2 microglial cells by suppressing TNF-*α* and IL-*β* through PI3K/akt/NF-kb signaling pathways. *Phytotherapy Research*.

[78] Young P. S., Ling J. M., Hye Y. E., Kim Y., Park G. (2018). Neochlorogenic acid inhibits against LPS-activated in-flammatory responses through up-regulation of Nrf2/HO-1 and involving AMPK pathway. *Environmental Toxicology and Pharmacology*.

[79] Kang O.-H., Choi J.-G., Lee J.-H., Kwon D.-Y. (2010). Luteolin isolated from the flowers of Lonicera japonica suppresses inflammatory mediator release by blocking NF-*κ*B and MAPKs activation pathways in HMC-1 cells. *Molecules*.

[80] Zhao J. W., Chen D. S., Deng C. S., Wang Q., Zhu W., Lin L. (2017). Evaluation of anti-inflammatory activity of compounds isolated from the rhizome of *Ophiopogon japonicas*. *BMC Complementary and Alternative Medicine*.

[81] Bi L.-Q., Zhu R., Kong H. (2013). Ruscogenin attenuates monocrotaline-induced pulmonary hypertension in rats. *International Immunopharmacology*.

[82] Tian Y. Q., Kou J. P., Li L. Z. (2011). Anti-inflammatory effects of aqueous extract from Radix Liriope muscari and its major active fraction and component. *Chinese Journal of Natural Medicines*.

[83] Ma L., Kou J. P., Huang Y. (2006). Effect of russaponin on adhesion of HL-60 to ECV304 cells. *Chinese Pharmacological Bulletin*.

[84] Dong J. X., Deng Y., Liu L. (2016). Effects of endophytic fermentation broth and host decoction, total flavonoids and total saponins from Gansu wild *Glycyrrhiza uralensis* on the secretion of inflammatory factors induced by LPS in RAW 264.7. *Journal of North Pharmacy*.

[85] Li X. H., Qi Y., Cai R. L. (2010). Anti-inflammatory mechanism of total saponins of *Glycyrrhiza uralensis*. *Chinese Journal of Experimental Formulaology*.

[86] Matsui S., Matsumoto H., Sonoda Y. (2004). Glycyrrhizin and related compounds down-regulate production of inflammatory chemokines IL-8 and eotaxin 1 in a human lung fibroblast cell line. *International Immunopharmacology*.

[87] Xie Y.-C., Dong X.-W., Wu X.-M., Yan X.-F., Xie Q.-M. (2009). Inhibitory effects of flavonoids extracted from licorice on lipopolysaccharide-induced acute pulmonary inflammation in mice. *International Immunopharmacology*.

[88] Kim Y. P., Lee E. B., Kim S. Y. (2001). Inhibition of prostaglandin E2 production by platycodin D isolated from the root of *Platycodon grandiflorum*. *Planta Medica*.

[89] Wang C., Schuller Levis G. B., Lee E. B. (2004). Platycodin D and D3 isolated from the root of *Platycodon grandiflorum* modulate the production of nitric oxide and secretion of TNF-*α* in activated RAW 264.7 cells. *International Immunopharmacology*.

[90] Yu W. Y., Zhu H. J. (2012). Study on pharmacological mechanism of *Platycodon grandiflorum* in treating bronchial asthma. *Chinese Medicine and Pharmacology*.

[91] Wang B., Gao Y., Zheng G. (2016). Platycodin D inhibits interleukin-13-induced the expression of inflammatory cytokines and mucus in nasal epithelial cells. *Biomedicine & Pharmacotherapy*.

[92] Han E. H., Park J. H., Kim J. Y., Chung Y. C., Jeong H. G. (2009). Inhibitory mechanism of saponins derived from roots of *Platycodon grandiflorum* on anaphylactic reaction and IgE-mediated allergic response in mast cells. *Food and Chemical Toxicology*.

[93] Park Y.-C., Jin M., Kim S.-H., Kim M.-H., Namgung U., Yeo Y. (2014). Effects of inhalable microparticle of flower of *Lonicera japonica* in a mouse model of COPD. *Journal of Ethnopharmacology*.

[94] Sun Q., Chen L., Gao M. (2012). Ruscogenin inhibits lipopolysaccharide-induced acute lung injury in mice: involvement of tissue factor, inducible NO synthase and nuclear factor (NF)-*κ*B. *International Immunopharmacology*.

[95] Cheng Z. H., Wu T., Li L. Z. (2005). Study on the fat-soluble chemical composition of traditional Chinese medicine *Ophiopogon japonicus*. *Journal of Chinese Pharmaceutical Sciences*.

[96] Qamar W., Khan R., Khan A. Q. (2012). Alleviation of lung injury by glycyrrhizic acid in benzo(a)pyrene exposed rats: probable role of soluble epoxide hydrolase and thioredoxin reductase. *Toxicology*.

[97] Zhang J. Y., Wei M. M., Chu X. (2012). Protective mechanism of Glycyrrhiza flavonoids on acute lung injury in mice. *Chinese Agricultural Science Bulletin*.

[98] Yao L., Zhang J.W., Meng Q.J. (2017). Study on lung injury induced by PM2.5 and the intervention of total saponins in Platycodon grandiflorum. *Chinese Journal of Information on Traditional Chinese Medicine*.

[99] Chen Q., Zhu M., Li Y. (2013). Interventional effect of *Platycodon saponins* on airway remodeling in mice with chronic bronchitis. *Journal of Anhui University*.

[100] Yao B., Zhang J. W., Meng Q. J. (2017). Study on the effects of PM2.5-induced lung injury and total saponins in *Platycodon grandiflorum*. *Chinese Journal of Information on Traditional Chinese Medicine*.

[101] Ishibashi H., Mochidome T., Okai J., Ichiki H., Shimada H., Takahama K. (2010). Activation of potassium conductance by ophiopogonin-D in acutely dissociated rat paratracheal neurons. *British Journal of Pharmacology*.

[102] Miyata T. (2000). Pathopharmacological evaluation of Bakumondo‐to(Mai‐Men‐Dong‐Tang) as a curative for chronic inflammatory airway disease. *Kampo Medicine*.

[103] Tang J., Qian H., Huang Q. (1999). Study on antiasthmatic and antiallergic effect of ophiopogon polysaccharide. *Chinese Journal of Modern Applied Pharmacy*.

[104] Hung T. M., Thu C. V., Dat N. T. (2010). Homoisoflavonoid derivatives from the roots of *Ophiopogon japonicus* and their *in vitro* anti-inflammation activity. *Bioorganic & Medicinal Chemistry Letters*.

[105] Han J., Zhao J., Tian W. Y. (2012). Effects of extra-intestinal transformation on the components and antitussive effects of *Glycyrrhiza uralensis*. *Journal of Anhui Medical University*.

[106] Yu T. F., Tian X. D., Li R. (1993). Antitussive and expectorant effects of *Glycyrrhiza uralensis* flavonoids, *Glycyrrhiza uralensis* extract and glycyrrhetinic acid. *Chinese Traditional Patent Medicine*.

[107] Wu Y. J. (1996). Study on antitussive, phlegm-eliminating and airway resistance-reducing effects of sodium glycyrrhetinate. *Journal of Lanzhou Medical College*.

[108] Zhu J. X., Zeng J. X., Zhang Y. M. (2015). Comparative study on the effects of cough and phlegm on *Platycodon grandiflorum* in different producing areas. *Modernization of Traditional Chinese Medicine and Materia-World Science and Technology*.

[109] Fang X. X., Huang B. T., Zeng J. X. (2016). Comparison of total saponins and platycodin D in *Platycodon grandiflorum* from different producing areas. *Chinese Journal of Experimental Traditional Medical Formulae*.

[110] Hu X. (2013). *Study on Auality Evaluation of Tetraploid Lonicera Japonica Medicinal Materials Combining Chemical Components with Acute Toxicity and Pharmacodynamics*.

[111] Pu H. L., Jiang H., Chen R. R. (2011). Toxicity study of *Lonicera japonica* and dried ginger on mice. *Practical Pharmacy and Clinical Remedies*.

[112] Lin Y. Y., Gu S. H., Song Y. L. (2019). Cytotoxicity of ophiopogon D' on H9c2 cells in cardiomyocytes. *China Pharmaceuticals*.

[113] Hu Y. P., Song J., Wang X. (2009). Study on genetic toxicity of ophiopogon decoction. *Chinese Journal of Information on Traditional Chinese Medicine*.

[114] Zhang M. Teratogenic effects of *Ophiopogon japonicus* decoction on rats.

[115] Zhao Z. H., Zhang L., Yong L. (2019). Safety evaluation of the upper part of the *Glycyrrhiza uralensis*. *Chinese Journal of Experimental Traditional Medical Formulae*.

[116] Chen X. C. (1984). The antagonism between *Glycyrrhiza uralensis* and euphorbiae kansui and its molecular complexes. *Chinese Traditional Patent Medicine*.

[117] Jin E. B., Jiang M. H., Huang Q. F. (1982). Pharmacological study on eighteen reversal of traditional Chinese medicine. *Chinese Traditional Patent Medicine*.

[118] Dai F. G., Luo R., Wang Y. G. (2005). Effect of euphorbiae kansui compatibility with *Glycyrrhiza uralensis* on CYP3A2 in rat liver. *Journal of the Fourth Military Medical University*.

[119] Jin X. J., Zhang M., Nie B. (2007). Acute and cumulative toxicity test of extract of *Platycodon grandiflorum*. *Feed Industry*.

